# Intra Uterine Forceps

**Published:** 1869-12

**Authors:** 


### Intra Uterine Forceps.

We have recently had our attention called to a new and very ingenious forceps, designed to be introduced while shut or closed into the uterine cavity, and, after being fully within, capable of being opened and rotated so as to grasp, with ease and certainty, a distended ovum, or foetus, or retained placenta, in cases of miscarriage, or any intra uterine growth, which may require removal. In its adaptation to these objects, it seems to us to so far surpass instruments formerly used for this purpose, as to render them comparatively worthless. Physicians constantly liable to have such cases fall under their care, should not fail to avail themselves of its advantages. This instrument is the invention of Dr. J. M. Blakesly, of Iowa City, Iowa, who is now offering it to the profession.

